# Molecular Genetic Diversity and Quantitation of Methanogen in Ruminal Fluid of Buffalo (*Bubalus bubalis*) Fed Ration (Wheat Straw and Concentrate Mixture Diet)

**DOI:** 10.1155/2013/980191

**Published:** 2013-06-05

**Authors:** K. M. Singh, A. K. Tripathi, P. R. Pandya, S. Parnerkar, R. K. Kothari, C. G. Joshi

**Affiliations:** ^1^P. G. Department of Genetics, ARIBAS, New V V Nagar, Anand, Gujarat 388121, India; ^2^Department of Animal Biotechnology, College of Veterinary Science and Animal Husbandry, Anand Agricultural University, Anand, Gujarat 388 001, India; ^3^Animal Nutrition Research Station, AAU, Anand, Gujarat 388001, India; ^4^Department of Microbiology, Christ College, Rajkot, Gujarat 360 005, India

## Abstract

High roughage diet causes more methane emissions; however, the total methanogen abundance is not influenced by roughage proportion. Technologies to reduce methane emissions are lacking, and development of inhibitors and vaccines that mitigate rumen-derived methane by targeting methanogens relies on present knowledge of the methanogens. In this work, we have investigated molecular diversity of rumen methanogens of Surti buffalo. DNA from rumen fluid was extracted, and 16S rRNA encoding genes were amplified using methanogen specific primer to generate 16S rDNA clone libraries. Seventy-six clones were randomly selected and analysed by RFLP resulting in 21 operational taxonomic units (OTUs). BLAST analysis with available sequences in database revealed sequences of 13 OTUs (55 clones) showing similarity with *Methanomicrobium* sp, 3 OTUs (15 clones) with *Methanobrevibacter* sp. The remaining 5 OTUs (6 clones) belonged to uncultured archaea. The phylogenetic analysis indicated that methanogenic communities found in the library were clustered in the order of Methanomicrobiales (18 OTUs) and Methanobacteriales (3 OTUs). The population of Methanomicrobiales, Methanobacteriales, and Methanococcales were also observed, accounting for 1.94%, 0.72%, and 0.47% of total archaea, respectively.

## 1. Introduction

Methanogens, members of the domain Archaea, fall within the kingdom euryarchaeota [1]. They are obligate anaerobes and can be unmistakably differentiated from other organisms since they all produce methane as a major catabolic end-product [2]. The most common species of methanogens isolated from the rumen are *Methanobrevibacter ruminantium*, *Methanomicrobium mobile, Methanobacterium formicicum, Methanobacterium smithii*, *Methanobacterium olleyae*, *Methanobacterium bryantii*, *Methanosarcina barkeri*, and *Methanoculleus olentangyi* [3–9]. The diversity of archaea found in the rumen has been recently reviewed [10–12].

Interest in methanogens from ruminants has resulted from the role of methane in global warming and from the fact that enteric methane emission is a major source of greenhouse gas in agriculture sector. Currently, India possesses the world's largest livestock population of 485 million, which accounts for 13% of the global livestock population (Intergovernmental Panel on Climate Change, 2001). It has 57% of the world's buffalo and 16% of the cattle population. Buffalo contributes to 42% of the total methane emission by livestock in India [13]. Reducing enteric methane emissions has been identified as one way of lowering global methane emissions. However, the effectiveness of any strategy that reduces greenhouse gas emissions and also increases production or nutritional efficiency will likely depend upon having an understanding of the numbers and/or distribution of methanogen species among ruminant livestock. In the present study, diversity analysis of methanogen consortium in ruminal fluid of buffalo (*Bubalus bubalis*) fed wheat straw and compound concentrate mixture was carried out.

## 2. Materials and Methods

### 2.1. Sampling and DNA Extraction

The permission of the Committee for the Purpose of Control and Supervision of Experiments on Animals (CPCSEA) was obtained prior to initiation of the study. The experiments were carried out on three young Surti buffaloes, approximately two years of age, which were reared at the Department of Animal Nutrition, College of Veterinary Science and A.H., Anand. All the animals were maintained under uniform feeding regime for minimum 30 days. The diet comprised wheat straw and compound concentrate mixture (20% crude protein, 65% total digestible nutrients). Approximately 500 mL of rumen fluid was collected 4 hrs after feeding [14]. About 100 mL rumen fluid was passed through four layers of cheese cloth to remove particulate matter. Remaining rumen fluid was stored at −80°C for further study. Total DNA was extracted separately by using a commercially available kit according to the manufacturer's instructions (QIAGEN Stool kit; QIAGEN, CA) and finally pooled all the DNA extracts. The total DNA was used as a template in PCR experiments to amplify 16S rDNA.

### 2.2. PCR Primers and Amplification

The 16S r DNA was amplified by PCR using metagenomic DNA as template and primers meth 86f (5′-ACAGGCCTAACACATGCAAGTC-3′) and meth1360r (5′-AGGGCGG(AT)GTGTACAAGGC-3′) [15]. A total of 25 *μ*L of reaction mixtures consisted of 10 pmol of each primer, 75–100 ng of template DNA, and 12.5 *μ*L of master mix (Fermentas, UK). The reaction mixtures were subjected to initial denaturation at 95°C for 5 minutes following 30 cycles of each denaturation at 94°C for 1 minute, annealing at 58°C for 45 seconds, and extension at 72°C for 1 minute with final extension at 72°C for 10 minutes using thermal cycler (ABI, USA). The anticipated product of approximately 1.2 kb was purified using Qiagen DNA Gel Extraction Kits (QIAGEN, CA) in accordance with the manufacturer's instructions.

### 2.3. Cloning, Screening, and RFLP

The purified PCR products were cloned in pTZR57T plasmid using the InstaT/A cloning kit (Fermentas, UK) according to the manufacturer's protocol and transformed into *Escherichia coli* DH5*α*. The blue and white colonies were screened on Luria-Bertani plates with ampicillin (100 *μ*g/mL), X-gal (20 mg/mL), and IPTG (100 mM). A total of 76 clones were examined from pooled metagenomes by colony PCR using vector specific primers M13f (5′-GTAAAACGACGGCCAG-3′) and M13r (5′-CAGGAAACAGCTATGAC-3′). Plasmids were extracted and amplified by PCR with primers M13f and M13r. The total 25 *μ*L volume of the reaction mixture contained 10 pmol of each primer, 12.5 *μ*L of master mix (Fermentas, UK), and 2.5 *μ*L plasmid DNA. PCR conditions were denaturation for 5 min at 94°C, 30 cycles of denaturation at 94°C for 1 min, annealing at 50°C for 1 min, extension at 72°C for 1 min, and a final extension at 72°C for 10 min. Aliquots (10 *μ*L) of all successfully recovered clones were digested with 0.5 U of HaeIII restriction endonuclease at 37°C overnight as described by [15]. The digested DNA was visualized after electrophoresis on 1.5% agarose gels. One of each RFLP profile was assumed to indicate a different sequence and each different RFLP profile was purified and used for sequencing.

### 2.4. Sequences and Phylogenetic Analysis

Sequencing performed for all the clones with an ABI Prism 310 Genetic Analyser (Applied Biosystems Inc., CA) using BigDye Terminator (version 3.1) cycle sequencing kit (ABI, USA) at the Animal Biotechnology Laboratory, AAU, Anand, Gujarat, India.

All reference sequences were obtained from the Genbank/EMBL [16]. Sequences from the current study were mainly trimmed to remove low-quality base calls from the start and end of DNA sequences and further analysed by the CHECK_CHIMERA program [17] to remove any chimera rDNA clone. The similarity searches for sequences were carried out by BLAST (http://www.ncbi.nlm.nih.gov/BLAST/Blast.cgi), and alignment was perform using CLUSTAL W (http://www.ebi.ac.uk/Tools/clustalw2/index.html). 

The phylogenetic relatedness was estimated using the neighbor-joining method [18]. All positions containing gaps and missing data were eliminated from the dataset (complete deletion option). One thousand bootstrap replications were performed to place the confidence estimates on the major groups resolved in the tree. The bootstrap consensus tree inferred from 1,000 replicates represents the evolutionary history of the sequences analyzed [19]. The phylogenetic analysis was carried out using MEGA software version 4.0 [20].

### 2.5. Real-Time PCR Analysis

Plasmid DNA containing the total archea, methanomicrobiales and methanobacteriales specific sequences, used as the standard DNA in real-time PCR, was obtained by PCR cloning with the specific primer sets already described [21]. After the confirmation of a single band of the correct size with respective pair of primers on an agarose gel, the PCR products were excised from the gel. The PCR products were purified using the Qiagen gel Purification Kit (Qiagen, CA) and then ligated into pTZR57T/A cloning vector (Fermentas, UK). The ligated products were transformed to competent *E. coli* DH5*α* cells by heat shock. Plasmids were purified from positive clones using a QIAprep spin miniprep kit (Qiagen, USA), and the plasmids containing the correct insert were screened out by PCR amplification with respective primer sets. Tenfold dilution series ranging from 108 to 10 copies were prepared for each target. Real-time PCR was performed with ABI system (ABI 7500). The Qiagen 2X SYBR Green master mix was used for PCR reaction. The optimal amplification conditions for each primer set were obtained with 10 pmol of each primer with the combination of annealing temperature and extension time as described by [21]. The 10-fold dilution series of the standard plasmid for the respective target was run along with the corresponding samples in duplicate. The copy numbers of 16S rRNA genes of targeted methanogens per mL rumen fluid were calculated using the following equation: (QM × C × DV)/(S × V), where QM is the quantitative mean of the copy number, C is the DNA concentration of each sample, DV is dilution volume of extracted DNA, S is the DNA amount (ng) subjected to analysis, and V is the rumen fluid volume subjected to DNA extraction [22]. In the reaction, nearly perfect linear regressions (r2 = 0.9930) to 0.9995 and slope (−3.3 to −4.5) were obtained between threshold cycle and quantities of standard for all targets, and data generated from the reaction were used for further analysis.

### 2.6. Nucleotide Sequence Accession Numbers

The nucleotide sequences of the 16S RNA gene from the representative clones (based on RFLP) were deposited in NCBI under the Accession nos. HM566228–HM566248.

## 3. Results

### 3.1. Comparison of Cloned Sequences with Sequences Deposited in Databases

A total of 76 clones from sample were screened and 21 sequences (OTUs) were generated based on PCR-RFLP patterns. Twenty-one sequences (OTUs) were subjected to similarity analysis using BLAST search [23] and SIMILARIY_RANK program [20]. In the library, a *Methanomicrobium* like clone accounted for almost 72.36% of the clones (55 of 76 clones), and 17.9% of the cloned sequences (15 clones) were aligned to *Methanobrevibacter* sp with 96%–98% identity. 7.89% of the cloned sequences (6 clones) Also, belonged to uncultured archaeon with 94%–98% similarity (Table 1).

### 3.2. Phylogenetic Analysis of Sequences and Quantitation of Methanogens

**Figure 1:**
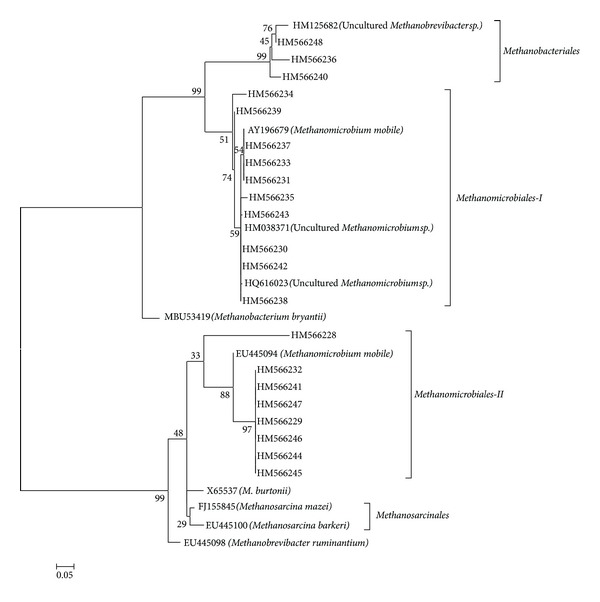
Phylogenetic relationships of partial 16S rDNA sequences of clones recovered from rumen samples of Surti buffalo. The tree was inferred by the neighbour joining method with 1,000 bootstrap replicates using the MEGA 4 tree building program. The scale bar represents 5% sequence divergence.

The results of phylogenetic analysis of sequences are shown in Figure 1. Rumen methanogens formed a tree mainly divided into clusters named Methanomicrobiales-II and Methanosarcinales, Methanomicrobiales-I, and Methanosarcinales. In cluster Methanomicrobiales-I, ten OTUs (26 clones) were phylogenetically placed within genus Methanomicrobia with only one species *Methanomicrobia mobile*. However Methanomicrobiales-I shows to be more related with Methanobacteriales. In the cluster Methanomicrobiales-II, eight OTUs (43 clones) were also phylogenetically placed within genus Methanomicrobia and the sequences were 94–98% identical to those of* Methanomicrobia mobile*. In cluster Methanobacteriales, three OTUs (7 clones) were phylogenetically affiliated to genus *Methanobrevibacter* sp with 96%–98% similarity (Table 1). Total archaea were detected with 7.23 × 10^7^copies per mL of ruminal fluid. The number of 16S rRNA gene copies of Methanomicrobiales, Methanobacteriales, and Methanococcales was detected with 1.4 × 10^6^, 5.2 × 10^5^, and 3.4 × 10^5^ copies per mL of ruminal fluid and accounting for 1.94%, 0.72%, and 0.47% of total archaea, respectively.

## 4. Discussion

Methanogens have been classified into more than 100 species distributed by more than 20 genera [24]. Interestingly, few methanogens have been isolated from the rumen. So far, cultured methanogens obtained from rumen have been assigned to *Methanobrevibacter ruminantium* [25], *Methanobrevibacter olleyae* [25], *Methanomicrobium mobile* [9, 26], and *Methanoculleus olentangyi* [27]. *Methanobacterium formicicum* [28] as well as *Methanosarcina* spp have also been cultured from the rumen [29]. However, in the present study, there were only a few genera identified in our library, which may be due to the ruminant host, diet, DNA extraction methods, or PCR primers used [30]. In addition, it is possible that the DNA from some methanogens could not be extracted or the extracted DNA of some species was too low for amplification. PCR amplification bias, primers, and geological distribution could be other reasons for the true diversity of the composition of rumen methanogens. Ramos et al. [31] and Nadais et al. [32] have developed a method for PCR analysis to characterize any microbial population in anaerobic sludge blanket (UASB) reactor sample based on the 16S r RNA-encoding genes and clones analysis using frequently cutting enzymes.

Methanogens in the present study, PCR-retrieved methanogenic 16S rRNA gene libraries, were established from the rumen fluid. The results showed that the rRNA gene clones generated in this study exhibited a high degree of sequence similarity to two methanogenic genus, *Methanomicrobium* and *Methanobrevibacter*. The most predominant species of methanogens in the study were related to the genera* Methanomicrobium*. This is an agreement with our previous report [33] of rumen methanogens of buffalo fed green fodder and concentrates, which indicates that diet composition does not affect the methanogen population. Predominance of sequences highly similar to those of the *Methanomicrobium* genus in the rumen of Indian buffalo has also been reported [34, 35]. In addition, Shin et al. [36] and Tajima et al. [37] have also reported the dominance of *Methanomicrobium* sp. rumen of cows and found that *Methanobrevibacter* sp. was either not detected or was not the dominant clade affiliated to genus *Methanobrevibacter* sp. However, in our study only few sequences were affiliated to genus *Methanobrevibacter*. In contrast, Whitford et al. [8] found that *Methanobrevibacter* sp. was the dominant archaea (58.5%) in the cow rumen, where *Methanosphaera* sp. (26.8%) and *Methanimicrococcus* spp. (14.6%) were also abundant. Wright et al. [30] found that *Methanobrevibacter* sp. dominated in feedlot cattle in two geographic locations in Canada while sheep in Australia and Venezuela shared very similar archaeal communities. Tatsuoka et al. [38] and Denman et al. [39] have reported the diversity of the methyl-coenzyme M reductase (*mcrA*) gene in the rumen of cattle and found that most of sequences belonged to *Methanobrevibacter* sp.

This study reveals the phylogenetic diversity of the rumen methanogens in Surti buffalo rumen by analyzing methanogenic specific16S rRNA gene sequences in a culture-independent manner. Sixteen out of the 21 sequences (OTUs) were associated with two methanogenic genera (Table 1): *Methanomicrobium* (13 sequences: HM566229, HM566230, HM566232, HM566234, HM566238, HM566239, HM566241, HM566242, HM566243, HM566244, HM566245, HM566246, and HM566247) and *Methanobrevibacter *(3 Sequences: HM566228, HM566240 and HM566248) (Figure 1). The remaining 5 sequences (OTUs) belonged to uncultured methanogenic archaeon. BLAST search showed that all sequences shared a degree of similarity ranging from 90% to 99% with ruminal archaeon 16S rDNA sequences deposited in NCBI GenBank database (Table 1).

In conclusion, the knowledge of the ruminal methanogen community is of critical importance for the development of strategies to mitigate rumen methane production. This study has revealed a predominance of *Methanomicrobium* in the rumen of Surti buffalo and indicates the need to better understand the diet dependent association study of methanogen with methane emission. Indeed on the basis of the information of these predominant methanogen species existing in their specific niche, this research provides basic information for further studies on elucidating the symbiotic relationship of methanogens and other microbes, the mechanism of methanogenesis, and the regulation of methane emissions in ruminants. Reducing enteric methane emissions is likely to be one of the key mitigation strategies for the reduction of greenhouse gas emissions in the agricultural sector.

## Figures and Tables

**Table 1:** Similarity values of 16S rRNA gene sequences retrieved from rumen of Surti buffalo.

Phylotypes (OTUs)	Clone no.	Accession no.	Genera	Nearest Taxa (Accession no.)	Identity (%)
ORFSBRM1	9	HM566228	*Methanobrevibacter* sp.	Uncultured *Methanobrevibacter *sp. (HM125682)	96
ORFSBRM2	10	HM566229	*Methanomicrobium *sp.	Uncultured *Methanomicrobium *sp. HQ616023.1	97
ORFSBRM3	1	HM566230	*Methanomicrobium *sp.	Uncultured *Methanomicrobium* sp. HQ616006.1	93
ORFSBRM4	1	HM566231	N/A	Uncultured archaeon clone sy-904231054-77-i GQ255495.1	94
ORFSBRM5	15	HM566232	*Methanomicrobium *sp.	Uncultured *Methanomicrobium *sp. HQ616023.1	98
ORFSBRM6	1	HM566233	N/A	Uncultured archaeon clone sy-904231051-84-i GQ255499.1	98
ORFSBRM7	5	HM566234	*Methanomicrobium* sp.	Uncultured *Methanomicrobium* sp. HQ616006.1	94
ORFSBRM8	2	HM566235	N/A	Uncultured methanogenic archaeon EU794774.1	90
ORFSBRM9	1	HM566236	N/A	Uncultured archaeon clone sy-904231051-40-i GQ255534.1	95
ORFSBRM10	1	HM566237	N/A	Uncultured archaeon clone sy-904231051-84-i GQ255499.1	98
ORFSBRM11	9	HM566238	*Methanomicrobium *sp.	Uncultured *Methanomicrobium *sp. HQ616023.1	98
ORFSBRM12	2	HM566239	*Methanomicrobium *sp.	Uncultured *Methanomicrobium *sp. HM038371.1	98
ORFSBRM13	2	HM566240	*Methanobrevibacter *sp.	Uncultured *Methanobrevibacter *sp. FJ919272.1	97
ORFSBRM14	3	HM566241	*Methanomicrobium *sp.	Uncultured *Methanomicrobium *sp. HQ616023.1	98
ORFSBRM15	2	HM566242	*Methanomicrobium *sp.	Uncultured *Methanomicrobium *sp. HQ616006.1	94
ORFSBRM16	2	HM566243	*Methanomicrobium *sp.	Uncultured *Methanomicrobium *sp. HQ616023.1	94
ORFSBRM17	1	HM566244	*Methanomicrobium *sp.	Uncultured *Methanomicrobium *sp. HQ616023.1	98
ORFSBRM18	2	HM566245	*Methanomicrobium *sp.	Uncultured *Methanomicrobium *sp. HQ616023.1	99
ORFSBRM19	2	HM566246	*Methanomicrobium *sp.	Uncultured *Methanomicrobium *sp. HQ616023.1	99
ORFSBRM20	1	HM566247	*Methanomicrobium mobile *	*Methanomicrobium mobile* M59142.1	99
ORFSBRM21	4	HM566248	Uncultured Methanobacteriales	Uncultured Methanobacteriales archaeon DQ402018.1	98

Total clones = 76.
